# Depression and anxiety symptoms among Arab/Middle Eastern American college students: Modifying roles of religiosity and discrimination

**DOI:** 10.1371/journal.pone.0276907

**Published:** 2022-11-03

**Authors:** Nadia N. Abuelezam, Sarah Ketchen Lipson, Sara Abelson, Germine H. Awad, Daniel Eisenberg, Sandro Galea

**Affiliations:** 1 William F. Connell School of Nursing, Boston College, Chestnut Hill, Massachusetts, United States of America; 2 Boston University School of Public Health, Boston, Massachusetts, United States of America; 3 University of Michigan School of Public Health, Ann Arbor, Michigan, United States of America; 4 Department of Educational Psychology, University of Texas at Austin, Austin, Texas, United States of America; Yale University, UNITED STATES

## Abstract

**Introduction:**

We examine mental health outcomes in a national sample of Arab/Middle Eastern college students using the Healthy Minds Study (HMS) from 2015–2018 and assess the modifying roles of religion and discrimination.

**Methods:**

HMS is an annual web-based survey administered to random samples of undergraduate and graduate students at participating colleges and universities. A total of 2,494 Arab/Middle Eastern and 84,423 white students were included in our sample. Our primary outcomes of depression and anxiety symptoms were assessed using the Patient Health Questionaire-9 (PHQ-9) and the Generalized Anxiety Disorder 7-item (GAD-7) scale, respectively. Survey-weighted logistic regression models were fit for each outcome using an Arab ethnicity indicator. Effect modification by religiosity and discrimination was examined by adding an interaction term to the model.

**Results:**

Odds of depression (adjusted odds ratio, AOR: 1.40, 95% CI: 1.24, 1.57) and anxiety (AOR: 1.41, 95% CI: 1.25, 1.60) were higher for Arab/Middle Eastern students than for white students. For Arab/Middle Eastern students, religiosity was a protective factor for both depression (AOR: 0.84, 95% CI: 0.79, 0.90) and anxiety (AOR: 0.91, 95% CI: 0.85, 0.97). Arab/Middle Eastern students who experienced discrimination had higher odds of depression (AOR: 1.41, 95% CI: 1.28, 1.56) and anxiety (AOR: 1.49, 95% CI: 1.36, 1.65) than those who had not.

**Discussion:**

Arab/Middle Eastern American college students are a vulnerable subgroup on college campuses experiencing a high burden of depression and anxiety symptoms which are dampened by religiosity and amplified by discrimination.

## Introduction

It is estimated that 3.7 million people living in the United States are of Arab and Middle Eastern descent, and this population is growing [1]. Individuals who have ethnic, linguistic, or cultural heritage from countries in the Middle East or North Africa and/or who speak Arabic are referred to as Middle Eastern or North African (MENA) Americans [1]. MENA Americans are a diverse group representing individuals from over 20 countries with different social and political contexts [2]. Yet, the population as a whole faces high levels of discrimination and racial-ethnic trauma in their daily lives in the United States. The terrorist attacks on September 11^th^ 2001 and the negative political and societal rhetoric that followed escalated the attention and discrimination directed at MENA Americans [3]. Further, recent reports show that hate crimes have increased against MENA Americans in recent years [4] and that national and international policies, like federal efforts to limit immigration from predominantly Muslim countries, have escalated these forces in MENA Americans’ lives [5].

Due to a lack of an ethnic/racial identifier for Arab and Middle Eastern groups in most studies, national and population-representative data on the mental health of MENA Americans are limited [6–8]. Public health research has not systematically identified MENA Americans in population-based studies in a way that enables a full assessment of the impact of discrimination on mental health [6]. Two national convenience sample surveys of Arab adults in the United States found high levels of depression and anxiety (50–62% and 25%, respectively) [9]. Other data from smaller samples [9–18] also suggest high levels of adverse mental health outcomes, like anxiety and depression, in this population.

Understanding the mental health needs of college students may provide important insights into the overall risk for depression and anxiety among MENA American adolescents and young adults in the United States. The representation of MENA individuals in colleges and universities may be higher than their proportional representation in the population. Results from the 2005 census show that 84% of the Arab American population aged 25 years and older hold a high school diploma and 41% hold at least a bachelor’s degree (compared to 80% and 24% in the United States population, respectively). Additionally, the age of onset of mental health issues aligns with age at college enrollment [19]. Prior work from our group suggests that MENA college students are at increased risk for poor mental health outcomes relative to their peers and are also less likely to access mental health treatment when in need [20]. However, the reasons for this elevated risk are relatively unknown.

Some potential differences between MENA college students and their peers are their experiences with religion and discrimination. Religion plays a large role in the cultural and traditional practices of MENA people and often acts as a tie to these immigrants’ ancestry and home countries [2]. The majority of individuals with Arab or Middle Eastern heritage living in the United States belong to one of two religious groups: Christians and Muslims [1]. Only a few studies have attempted to understand the role of religiosity on mental health outcomes for MENA Americans, despite a large literature on the relationship between religiosity and health in other populations [21, 22]. There is evidence, however, that religion may influence health behaviors and how MENA people are treated in ways that could influence mental health. Religiosity may serve to strengthen coping and improve psychological well-being, as was found in a study of Arab American adults from 2009 [10]. However in other research, religiosity predicted discrimination among Muslims and the interaction of religiosity and discrimination predicted psychological distress [15].

Religiosity in a college context has been found to be important, particularly for students with minority ethnic background [23]. In a study of 413 undergraduates from a Southwestern university, the authors found that ethnicity moderated the relationship between religiosity and mental health outcomes [23]. Religious African Americans had better mental health outcomes than religious European Americans [23]. In a web-based study of Arab college students, religiosity was predictive of lower risk of depression for Muslims but not for Christians, in contrast to studies among Arab American adults [24].

Little work has been done to assess the burden and impact of experiencing discrimination among MENA college students or MENA Americans in general. Hate crimes and bias incidents have been increasing on college campuses nationwide [25, 26]. In 2015, violence against Arab American Muslim college students occurred in Chapel Hill, North Carolina when three Muslim Arab Americans were killed by a gunman on account of their religious affiliation [4]. In studies of MENA adults, Muslim MENA Americans report higher levels of discrimination than Christian MENA Americans [11, 12, 27]. Work in adult MENA populations, in a number of contexts, have found a relationship between ethnic discrimination and poor mental health outcomes [11, 12, 28]. In a study of Iraqi adolescents aged 12–17 in Michigan, investigators found that the adolescents reported high exposure to interpersonal discrimination, media discrimination, and government or institutional discrimination [29]. Each of these forms of discrimination was highly correlated with major depression symptoms, risk of suicide, and other mental health conditions [29]. Fifty-eight percent of the sample reported more than 20 total macroaggressions in the past year [29].

There are, therefore, three scientific gaps we addressed in our analysis: First, we estimated the prevalence and odds of depression and anxiety among MENA and non-MENA white college students between 2015 and 2018; second, we determined if Arab ethnicity was associated with these symptoms among college students; third, we quantified the role of religiosity and discrimination as modifiers of the relationship between Arab ethnicity and mental health.

## Methods

### Sample

The Healthy Minds Study (HMS) is a web-based survey administered annually to random samples of undergraduate and graduate students at participating colleges and universities. The survey measures mental health status, service utilization, and related factors, and has an extensive demographics section. Colleges and universities elect to participate in HMS; there are no exclusion criteria for institutional enrollment. In the present study, we use data from 2015–2018, which include students from 124 campuses in the United States. The institutional sample is geographically and institutionally diverse (63 public, 61 private). A random sample of 4000 students at each institution (or all students at smaller campuses) over the age of 18 are invited to participate via email. Students must provide electronic consent before entering the survey. Survey response rates were 27% for the 2015–2016 cycle and 24% for the 2016–2017 and 2017–2018 cycles [30–32]. HMS methods have been described in more detail in previous publications [20, 33].

The study was approved by a central Institutional Review Board (Advarra, HUM00100169) and further approved at each of the participating campuses. The study is also covered by Certificate of Confidentiality from the National Institutes of Health. Consent was documented electronically. Students clicked on a link to the survey in the recruitment email and were presented with an informed consent page. They were informed that participation was voluntary. They had to agree to the terms of the consent form before entering the survey.

### Ethnicity

Students were asked to identify their race/ethnicity using the following question: “*What is your race/ethnicity*? *(select all that apply)*” with the following response categories: “*African American/Black*, *American Indian or Alaska Native*, *Asian American/Asian*, *Hispanic/Latino/a*, *Native Hawaiian or Pacific Islander*, *Middle Eastern/Arab/Arab American*, *White*, *or Self-identify*.” A student was considered to be MENA American if they selected “Middle Eastern/Arab/Arab American” from the list. Despite the fact that the race/ethnicity question in HMS did not explicitly ask about North African status, we use the MENA nomenclature to describe the results of this to be consistent with prior literature. Students who selected more than one race/ethnicity category with the “Middle Eastern/Arab/Arab American” category were considered to be MENA American. Students who did not select the “Middle Eastern/Arab/Arab American” option but did select the “White” option (whether solely or with another response category) were considered non-MENA white and included in the comparison group for this analysis. We seek to compare MENA American students to non-MENA white students, as the majority of MENA individuals have been found to select “White” on standard racial/ethnicity surveys without a dedicated MENA indicator [34].

### Primary outcomes

We examine two binary outcomes (dependent variables) related to mental health symptoms: (1) Symptoms of depression are assessed using the Patient Health Questionaire-9 (PHQ-9) [35, 36] which has been validated previously among diverse populations [37–39]. We used a standard cutoff of > = 10 to indicate a positive screen for depression among respondents. (2) Symptoms of anxiety are assessed using the Generalized Anxiety Disorder 7-item (GAD-7) scale [40]. We used a standard cutoff of > = 10 to indicate a positive screen for anxiety. The scale has been used and validated in diverse populations [41]. It is important to note that both the PHQ-9 and GAD-7 are screening tools and are not diagnostic tools.

### Covariates

The following indicators were used as covariates in the analysis and were compared for MENA and non-MENA American students: age (<21 vs. 21+), degree level (undergraduate vs. graduate), gender (male/female/gender minority), sexual orientation (heterosexual vs. lesbian/gay vs. bisexual/questioning/other), first generation college student (first generation vs. other), and citizenship (United States citizen vs. non-citizen).

### Modifiers

Effect modifiers were examined for the relationship between Arab ethnicity and mental health outcomes. Two effect modifiers were considered for this analysis: religiosity and discrimination. Religiosity was assessed by the following question: “*How important is religion in your life*?” with response options: *Very important*, *Important*, *Neutral*, *Unimportant*, *and Very unimportant*. Students who indicated that religion was *Very important* or *Important* were considered to have high religiosity. To assess discrimination, students were asked: “*In the past 12 months*, *how many times have you been treated unfairly because of your race*, *ethnicity*, *gender*, *sexual orientation*, *or cultural background*?” with response options: *Never*, *Once in a while*, *Sometimes*, *A lot*, *Most of the time*, *Almost all the time*. Students who answered with *Sometimes*, *A lot*, *Most of the time*, or *Almost all the time* were considered to have experienced discrimination in the past 12 months.

### Analysis

Sample probability weights were constructed to adjust for potential differences between responders and non-responders. Information about gender, race/ethnicity, academic level, and grade point average were obtained for the full samples invited to the survey from participating institutions. Weights (the reciprocal of the estimated probability of response) were constructed from these data using logistic regression models for the likelihood of response for each variable.

Covariates, primary modifiers, and outcomes were examined and stratified by ethnicity, and chi-square statistics were used to compare MENA American and non-MENA white students on these values. Survey-weighted logistic regression models were fit for each outcome using an MENA ethnicity indicator in unadjusted analyses, and including covariates in adjusted analyses. Odds ratios (OR) and adjusted odds ratios (AOR) were estimated for the ethnicity indicator (independent variable). Effect modification was examined by adding an interaction term examining the interactive effects of the effect modifiers and the MENA indicator of interest. All analyses were run using SAS v9.4 and applied survey non-response weights.

## Results

### Sample description

A total of 2,494 MENA American students and 84,423 non-MENA white students were identified for the analysis with non-missing values on age, gender identity, sexual orientation, first generation college student status, the depression scale, and the anxiety scale. Among MENA American students, 40.6% reported a race/ethnicity in addition to “Middle Eastern/Arab/Arab American” (Table 1). Among non-MENA white students, 11.4% reported a race/ethnicity in addition to “White” (Table 1). Fewer MENA American students were aged 21 or younger (49.7 vs. 60.3%, p<0.05) and undergraduates (64.5 vs. 70.6%, p<0.05) than non-MENA students (Table 1). In both groups, just over 50% identified as female and heterosexual (Table 1). Only 75.3% of MENA American students were United States citizens compared to 97.7% of non-MENA students. The majority of MENA American students identified as Muslim (43.7%) or Christian (22.6%) and non-MENA students as Christian (54.8%).

**Table 1:** Description of the Healthy Minds Study sample of Middle Eastern/Arab American (MENA) college students and non-MENA white college students.

	MENA students N = 2,494	non-MENA white students N = 84,423	*significant diff*?
**Demographics, N (weighted %)**			
Age (21 or younger)	1153 (49.7)	48819 (60.3)	*
Undergraduate	1428 (64.5)	57073 (70.6)	*
Gender			*
*Male*	917 (45.5)	26087 (40.3)	
*Female*	1522 (52.1)	56429 (56.8)	
*Gender minority*	55 (2.4)	1907 (2.8)	
Self-identified race/ethnicity (not exclusive)			
*White*	851 (32.6)	84423 (100)	
*Black*	92 (4.2)	1338 (1.8)	
*Hispanic*	142 (5.5)	4520 (5.3)	
*American Indian or American Native*	40 (1.8)	1361 (1.8)	
*Asian or Pacific Islander*	147 (6.1)	2848 (3.1)	
*Other*	69 (3.0)	597 (0.8)	
Sexual orientation			*
*Heterosexual*	2105 (83.9)	70210 (82.0)	
*Lesbian/Gay*	200 (7.9)	7977 (10.2)	
*Bisexual/Questioning/Other*	189 (8.1)	6236 (7.9)	
First generation	648 (28.4)	22352 (30.0)	
Citizen	1862 (75.3)	82296 (97.7)	*
Religion (not exclusive)			*
*Christian*	645 (22.6)	51077 (54.8)	
*Muslim*	1081 (43.7)	279 (0.3)	
*Atheist/Agnostic*	461 (15.6)	22104 (23.4)	
*Other*	484 (16.7)	17457 (20.0)	
*Missing*	207 (6.9)	5204 (4.8)	
**Mental Health Symptoms, N (weighted %)**			
Depression (PHQ-9> = 10)	829 (36.0)	24693 (31.5)	*
Anxiety (GAD-7> = 10)	734 (31.9)	22166 (27.5)	*
**Modifiers, N (weighted %)**			
Experienced discrimination in the past 12 months	261 (11.3)	3655 (5.1)	*
Religiosity (important)	652 (27.9)	12820 (15.4)	*

* Denotes a statistically significant difference between MENA and non-MENA white students at the p<0.05 level using a chi-squared statistic.

### Depression and anxiety symptoms

Depression symptoms, measured through the PHQ-9 scale, were more common among MENA American students than non-MENA white students (36.0 vs. 31.5%, p<0.05) (Table 1). Odds of depression symptoms were higher for MENA American students than for non-MENA white students (adjusted odds ratio, AOR: 1.40, 95% CI: 1.24, 1.57) (Table 2). Anxiety symptoms were more common among MENA American students than non-MENA white students (31.9 vs. 27.5%, p<0.05) (Table 1). Odds of anxiety symptoms, measured by GAD-7, were higher for MENA American students than non-MENA white students (AOR: 1.41, 95% CI: 1.25, 1.60) (Table 2).

**Table 2:** Results of unadjusted and adjusted logistic regression for depression and anxiety in the Healthy Minds Study sample comparing Arab/Middle Eastern American (MENA) college students to non-MENA white college students.

	*MENA vs*. *non-MENA white*	
	Unadjusted OR (95% CI)	Adjusted* OR (95% CI)
**Mental Health Symptoms**		
Depression (PHQ-9)	1.24 (1.12, 1.38)	1.40 (1.24, 1.57)
Anxiety (GAD-7)	1.26 (1.13, 1.40)	1.41 (1.25, 1.60)

*Adjusted for age (< = 21/22+), gender (male/female/other), sexual orientation (hetero/gay/other), first generation (yes/no), citizen (yes/no)

### Effect modification

More MENA American students reported that religion was important to them than non-MENA white students (27.9 vs. 15.9%, p<0.05). For MENA American students, religiosity was associated with lower odds for all primary outcomes (Table 3). Non-MENA white students who did not think religion was important had lower odds of depression (AOR: 0.79, 95% CI: 0.74, 0.85) and anxiety symptoms (AOR: 0.79, 95% CI: 0.73, 0.84) than MENA American students who did not think religion was important.

**Table 3:** Results of adjusted logistic regression models incorporating an interaction term to examine the modifying roles of religion and discrimination on Arab ethnicity and mental health outcomes (depression and anxiety). Models were adjusted for age (< = 21/22+), gender (male/female/other), sexual orientation (hetero/gay/other), first generation (yes/no), citizen (yes/no).

	MENA students, AOR (95% CI)	non-MENA white students, AOR (95% CI)
**Depression (PHQ-9)**		
Experienced discrimination in past 12 months	1.41 (1.28, 1.56)	1.27 (1.11, 1.44)
No discrimination in past 12 months	1.00 (ref)	0.93 (0.84, 1.03)
Religiosity (important)	0.84 (0.79, 0.90)	0.61 (0.56, 0.66)
Not important (religiosity)	1.00 (ref)	0.79 (0.74, 0.85)
**Anxiety (GAD-7)**		
Experienced discrimination in past 12 months	1.49 (1.36, 1.65)	1.18 (1.04, 1.33)
No discrimination in past 12 months	1.00 (ref)	0.87 (0.79, 0.96)
Religiosity (important)	0.91 (0.85, 0.97)	0.65 (0.60, 0.71)
Not important (religiosity)	1.00 (ref)	0.79 (0.73, 0.84)

ref = reference

More MENA American students experienced discrimination in the year prior to the survey than non-MENA white students (11.3 vs. 5.1%, p<0.05). Among MENA American students, those who experienced discrimination over the past year had increased odds of depression (AOR: 1.41, 95% CI: 1.28, 1.56) and anxiety (AOR: 1.49, 95% CI: 1.36, 1.65) symptoms when compared to MENA students who had not experienced discrimination (Table 3). Experiencing discrimination was also associated with increased odds of depression (AOR: 1.27, 95% CI: 1.11, 1.44) and anxiety (AOR: 1.18, 95% CI: 1.04, 1.33) symptoms among non-MENA white students when compared to MENA students who had not experienced discrimination.

## Discussion

In a well-established population study of college students in the United States, we found a higher burden of depression and anxiety symptoms among MENA American college students when compared to non-MENA white college students. This result is particularly striking given that MENA individuals are regularly categorized as non-Hispanic white on standard racial/ethnic forms (including on university campuses) suggesting that racial and ethnic reporting standards are masking disparities. There are limited population-based nationally representative studies examining the prevalence of mental health symptoms in this population to compare to, because of the standards in racial/ethnic reporting questions [6]. Among national convenience sample surveys of Arab adults in the United States, between 50–62% had Center for Epidemiologic Studies Depression Scale (CES-D) scores indicating depression [10] and 25% of respondents reported moderate to severe anxiety (Beck Anxiety Scale) [9]. The prevalence of depression (36.0%) symptoms among MENA American college students in our sample was lower than these national surveys but the prevalence of anxiety symptoms (31.9%) was higher (noting different measures/scales). These differences could be due to a number of factors including differences in measures being used, the sampling approach, and the time period in which the studies were conducted.

Based on prior work in this population, we hypothesized that differences in depression and anxiety symptoms between MENA and non-MENA students could be attributed to the modifying roles of religiosity and discrimination. Religiosity among MENA American students was associated with lower odds of both primary outcomes. MENA American students who did not think religion was important had higher odds of depression symptoms (PHQ-9) and anxiety symptoms (GAD-7) than non-MENA white students who did not think religion was important. The relationship between religiosity and mental health outcomes has been debated over the last 50 years in the public health literature [21, 42]. A recent systematic review found that in studies aiming to examine the relationship between religiosity and depression, 61% found that greater religiosity led to lower depression while 32% found no association [43]. For anxiety, 49% found that greater religiosity led to lower anxiety while 40% found no relationship. While our study found reduced odds of depression and anxiety symptoms for religious MENA American students compared to non-religious MENA students, MENA students who do not believe that religion is important had higher odds of depression and anxiety symptoms than non-MENA white students who are not religious.

A large proportion of MENA American students in our study experienced discrimination. Experiencing discrimination in the past year modified the relationship between Arab ethnicity and mental health outcomes, with MENA students experiencing discrimination having greater odds of depression and anxiety symptoms than MENA students who did not experience discrimination in our study. The Model of Cumulative Racial-Ethnic Trauma among Americans of Middle Eastern and North African Descent highlights the important role of both macro- and micro-level factors that lead to cumulative trauma in this population [3]. Macro-level forces include historical trauma, national context, and institutional discrimination while micro-level factors include interpersonal discrimination/microaggressions and lack of identity recognition. In a study after September 11^th^ 2001 among Arab Americans (N = 342), 63% reported discrimination or bad treatment in the workplace and 26% reported having been called a name, while 11% reported physical attacks or other traumatic events associated with racism [10]. In another study conducted in the Detroit area using face-to-face interviews of over 1000 Arab Americans, 25% reported discrimination related to race, ethnicity, or religion [12]. Authors found that Arabs reporting bad experiences related to ethnicity had worse Kessler Psychological Distress scores [12]. Data from Michigan showed that among Arab Americans who perceived they were discriminated against, they had increased odds of psychological distress, especially among males [14]. In in-depth interviews with 30 Muslim women of Arabic heritage, 63% reported discrimination and 25% reported clinical depressive symptoms [17].

This analysis has a few limitations that should be considered in interpreting the results. First, the racial and ethnic identifiers on the HMS survey did not explicitly name North African ethnicity. This differs from prior work in this field which highlights health risks in MENA population. Our work falls within the definition of MENA. While we might expect North Africans to respond as Arabs in our study [34], we were not able to validate this selection. Therefore, we caution against using this work to generalize to all MENA populations and suggest that this work can most fruitfully point to further work that can more explicitly assess MENA populations around the questions at hand. Additionally, the heterogeneity in this group is quite high which may limit our ability to generalize our findings. Second, the response rates for MENA students within the online HMS are unknown. It is unclear, therefore, if MENA students are less likely to respond to the survey than students from other racial or ethnic sub-groups. Although the sample probability weights adjusted for potential non-response bias related to other demographic characteristics, non-response specific to MENA students could lead to bias in survey results that is unmeasurable. Third, the measures for depression and anxiety symptoms were self-reported and do not reflect clinical diagnosis. Fourth, a factor known to be important to mental health among other minority groups is acculturation status. While we have adjusted for some variables that may reflect acculturation status (citizenship), we have not fully accounted for acculturation in our analysis. Fifth, our measure for discrimination does not ask exclusively about unfair treatment due to race or ethnicity. It is unclear if MENA students are reporting ethnic or racial discrimination or discrimination related to some other identity they hold but future work through HMS will incorporate survey items that aim to understand discrimination in more detail. Sixth, the mediators in this analysis (religiosity and discrimination) were measured using a single question each. Further context may have helped elucidate the proposed mechanisms. For example, we did not examine differences between Muslim and Christian MENA college students but plan to do this in future analyses given previous comparisons made in the literature.

Despite these limitations, we have conducted the largest study to date on MENA American college students’ mental health. Our findings indicate that MENA American college students are a vulnerable subgroup on American college campuses. College campuses should undertake efforts to revise standard racial and ethnic survey measures to better capture the needs of this minority population. More effort should be made by college campuses to ensure inclusive and safe environments for all students from all backgrounds with a special focus on those with Arab and Middle Eastern heritage. Ensuring MENA college students have appropriate and culturally sensitive mental health care should be a priority for college campuses given the potential for both racial-ethnic trauma and poor mental health outcomes. This field would benefit from prospective studies examining how depression and anxiety symptoms in MENA American populations change over time with changes to the social environment. Work should be undertaken to ensure and evaluate whether college campuses provide safe spaces for MENA American students despite a turbulent time in our nation’s history.
